# Neuromuscular adaptations to resistance training in elite versus recreational athletes

**DOI:** 10.3389/fphys.2025.1598149

**Published:** 2025-06-09

**Authors:** Sumaira Aslam, Jean De Dieu Habyarimana, Shi Yong Bin

**Affiliations:** Department of Physical Education, Henan University, Kaifeng, China

**Keywords:** motor unit recruitment, hypertrophy, periodization, fatigue management, athletic development

## Abstract

Neuromuscular adaptations to resistance training drive strength and performance improvements, but differences between elite and recreational athletes remain underexplored. Understanding the underlying mechanisms can refine training approaches and enhance athletic development. This review synthesized findings from the past decade regarding how training status, age, sex, and genetics influence neuromuscular adaptations to resistance training, identified key gaps in the literature, and provided practical recommendations for tailoring training to different athletic levels. This critical review synthesized evidence on neuromuscular adaptations to resistance training, focusing on muscle hypertrophy, architectural changes, motor unit recruitment, neural drive, fiber-type transitions, and genetic influences. Methodological limitations and gaps were highlighted, with a focus on elite versus recreational populations. Muscle hypertrophy and strength gains occur rapidly in novices but plateau in advanced athletes, requiring more complex stimuli. Neural adaptations, including improved motor unit synchronization and reduced antagonist co-contraction, distinguish elite from recreational athletes. Genetic predispositions and training history further modulate adaptations. Fatigue, recovery, and injury risk differ between groups, underscoring the need for tailored monitoring and recovery strategies. Research gaps include inconsistent methodologies, limited elite athlete data, and underrepresentation of female cohorts. Future studies should integrate neurophysiological tools and long-term designs to clarify these mechanisms. Effective training requires adjusting intensity and volume based on an athlete’s training status. Foundational strength programs benefit youth, while elite athletes require periodization and advanced methods. Policy-level support for supervised resistance training in youth can enhance performance and injury resilience. Addressing these insights can optimize training outcomes across athletic levels.

## 1 Introduction

Neuromuscular adaptations encompass the physiological changes in muscle structure and neural function that occur in response to resistance training (Blagrove et al., 2024; Santos et al., 2023). Resistance training remains a fundamental component of athletic development, driving improvements in strength, power, muscle hypertrophy, and neuromuscular efficiency (Cross et al., 2018). These adaptations are essential for enhancing athletic performance, enabling athletes to generate greater force, increase power output, and sustain performance under fatigue (Cross et al., 2018; Tomažin et al., 2021).

The primary goal of neuromuscular adaptations to resistance training is to improve strength, power, and movement coordination by enhancing the nervous system’s capacity to recruit and activate muscle fibers more effectively, thereby increasing force production and movement efficiency (Sale, 1988; Bandy et al., 1990). However, the precise neural mechanisms underlying enhanced force generation and motor control in athletes remain poorly understood (Škarabot et al., 2021a; Feuerbacher et al., 2025; Walker, 2021). Elite athletes often face diminishing returns and elevated injury risks due to training saturation and the heightened intensity required to elicit further adaptations (Gabbett, 2016; Jones et al., 2017). In contrast, novice athletes experience inconsistent improvements and greater injury susceptibility, primarily due to underdeveloped motor control and inadequate recovery strategies (Charest and Grandner, 2022). Emerging methodologies, such as velocity-based training and eccentric overload, present promising avenues for enhancing training outcomes, but their practical application remains inconsistent or suboptimal. This gap between theoretical insights and applied practice underscores the need for a more comprehensive and individualized approach to resistance training that integrates both physiological and psychological dimensions of adaptation.

Recent research has increasingly focused on the influence of training status, age, and gender on neuromuscular adaptations (Santos et al., 2023; Tumkur Anil Kumar et al., 2021; Jones et al., 2021), including motor unit recruitment, firing rates, hypertrophy, and coordination. Distinct responses between elite and recreational athletes reflect differences in training volume, intensity, and physiological capacity. However, the interaction of training status, age, and gender remains insufficiently explored, with many studies examining isolated variables or generalizing findings across populations (Kjølhede et al., 2015; Conlon et al., 2017; Akagi et al., 2020). This review therefore synthesizes findings from the past decade on neuromuscular adaptations to resistance training, examining differences between elite and recreational athletes, underlying muscular and neural mechanisms, training methodologies, recovery strategies, and practical recommendations for optimizing athletic development and injury prevention.

### 1.1 Search strategy

Although this review adopts a narrative approach, methodological rigor was maintained through a structured literature search to ensure transparency and relevance. A comprehensive search was conducted across four major academic databases: PubMed, Scopus, Web of Science, and SportDiscus. The search strategy utilized a combination of keywords and Boolean operators, including: “neuromuscular adaptations,” “fatigue management,” “elite athletes,” “recreational athletes,” “muscle hypertrophy,” “neural drive,” “resistance training,” and “training periodization.” The search focused on literature published between 2015 and 2024 to capture recent empirical advances in the field.

Studies were included if they met the following criteria: (1) peer-reviewed original research articles; (2) focused on neuromuscular adaptations to resistance training; (3) involved comparative analyses of elite and recreational athletes; (4) reported quantitative outcomes such as electromyographic activity, strength gains, fiber-type shifts, or muscle architectural variables; and (5) were published in English. Exclusion criteria encompassed: animal studies, non-English publications, theoretical articles or reviews lacking primary data, and studies without clearly defined training status classifications, i.e., elite vs. recreational.

The initial database search identified 1,250 records. After title and abstract screening, articles were assessed against the inclusion/exclusion criteria. Eighty-five full-text articles were reviewed in detail, with 22 studies ultimately meeting all criteria for inclusion in the synthesis. These studies formed the evidential basis for examining trends, discrepancies, and implications across training levels (see Table 1 for a summary of included studies).

**TABLE 1:** Overview of studies on neuromuscular adaptation and resistance training in elite vs. recreational athletes.

Study title	Authors	Objective	Methodology	Participants	Key findings
The Role of Musculoskeletal Training During Return to Performance	Blagrove, R.C., et al.	To understand neuromuscular changes during return from energy deficiency in sport	Cross-sectional study, examined musculoskeletal recovery and adaptations post-energy deficiency in athletes	Energy-deficient athletes returning to performance	Musculoskeletal recovery from energy deficiency requires tailored strength protocols
Long-Term Neurophysiological Adaptations to Strength Training	Santos, P.D.G., et al.	To analyse neurophysiological changes in strength-trained athletes over time	Systematic review, focusing on long-term adaptations with cross-sectional comparisons	Strength-trained athletes (long-term)	Long-term strength training induces significant neurophysiological adaptations
Training at Maximal Power in Resisted Sprinting	Cross, M.R., et al.	To assess optimal load for maximal power in resisted sprinting	Pilot study with team sport athletes, load variations on sprinting	Team sport athletes involved in sprint training	Optimal load for maximal sprinting power identified
Neuromuscular Adaptations after an Altitude Training Camp	TomaÅ¾in, K., et al.	To evaluate neuromuscular adaptations to altitude training in elite athletes	Cross-sectional study with judo athletes, focused on muscle hypertrophy and neural efficiency	Elite judo athletes undergoing altitude training	Altitude training positively impacts muscle and neural adaptation in elite athletes
Neuromuscular adaptations to different set configurations during a periodized power training block	Harris, D.M., Oranchuk, D.J., Latella, C	To assess neural and muscular adaptations to periodized power training in elite junior judokas	Longitudinal study with resistance training intervention in elite junior athletes	Elite junior judokas undergoing periodized power training	Periodized power training enhances neural and muscular adaptations in elite athletes
Greater neural adaptations following high vs. low-load resistance training	Jenkins, N.D., et al.	To compare neural adaptation responses in high-load vs. low-load training	Experimental design, comparing neural adaptations between high-load and low-load training in recreational athletes	Recreational athletes with high-load and low-load training comparisons	High-load training induces superior neural adaptations compared to low-load training
Resistance training variables for optimization of muscle hypertrophy	BernÃ¡rdez-VÃ¡zquez, R., et al.	To evaluate different resistance training variables influencing muscle hypertrophy in athletes	Systematic review and meta-analysis on resistance training protocols and hypertrophic responses	Resistance-trained athletes undergoing various resistance training protocols	Key variables in resistance training determine hypertrophic outcomes
Neural adaptation to resistance training	Sale, D.G.	To investigate neural adaptation mechanisms during resistance training	Cross-sectional study, focusing on neural efficiency changes and muscle performance	Strength-trained individuals, comparing different resistance training models	Resistance training promotes neural efficiency and muscular gains
Adaptations to endurance and strength training	Hughes, D.C., Ellefsen, S., Baar, K	To explore differences in adaptation between endurance and strength training modalities	Comparative study on endurance vs. strength training effects in recreational athletes	Endurance vs. strength training athletes	Endurance and strength training lead to distinct neuromuscular adaptations
Greater Strength Gains after Training with Accentuated Eccentric than Traditional Isoinertial Loads	Walker, S., et al.	To compare strength gains between accentuated eccentric training vs. traditional strength training	Experimental study involving strength-trained men, comparing eccentric load variations	Strength-trained athletes in eccentric load training studies	Accentuated eccentric training leads to better strength gains than traditional methods
Muscle adaptations to heavy-load and blood flow restriction resistance training methods	May, A.K., et al.	To evaluate muscle adaptations to heavy-load vs. blood flow restriction training	Comparative study on muscle hypertrophy and strength between high-load and blood flow restriction training methods	Resistance-trained individuals undergoing blood flow restriction training	Blood flow restriction enhances hypertrophy and strength in trained athletes
Correlation between load volume and indicators of adaptive body changes	Chernozub, A., et al.	To correlate load volume with muscle adaptations in untrained young men	Cross-sectional study evaluating training load and physiological changes during resistance training in untrained athletes	Untrained young men undergoing resistance training	Load volume positively correlates with muscle adaptation in untrained athletes
The Effects of Resistance Training on Sport-Specific Performance of Elite Athletes	Makaruk, H., et al.	To assess the impact of resistance training on sport-specific performance in elite athletes	Meta-analysis of resistance training effects on physical performance measures in elite athletes	Elite athletes in various sport-specific training regimens	Resistance training significantly improves sport-specific performance in elite athletes
What performance characteristics determine elite vs. nonelite athletes in the same sport?	Lorenz, D.S., et al.	To determine performance characteristics that differentiate elite from non-elite athletes	Comparative study across different levels of athletes in the same sport	Elite vs. non-elite athletes in the same sport	Elite athletes outperform non-elites due to improved neuromuscular efficiency
Resistance training prescription for muscle strength and hypertrophy in healthy adults	Currier, B.S., et al.	To review the optimal resistance training prescription for muscle strength and hypertrophy in adults	Systematic review and Bayesian meta-analysis	Adults undergoing prescribed resistance training for strength and hypertrophy	Systematic resistance training prescription leads to optimal hypertrophic and strength outcomes
Effects of strength training on physical fitness and sport-specific performance in recreational, sub-elite, and elite rowers	Thiele, D., et al.	To evaluate the effects of resistance training on performance across recreational, sub-elite, and elite rowers	Systematic review and meta-analysis	Rowers at different training levels (recreational, sub-elite, elite)	Resistance training improves performance in recreational, sub-elite, and elite rowers
The impact of training volume and intensity on muscle hypertrophy	Mangine, G.T., et al.	To determine how volume and intensity influence hypertrophy in resistance-trained men	Randomized controlled trial comparing different volumes and intensities in resistance training for hypertrophic outcomes	Resistance-trained men of various volumes and intensities	Training volume and intensity directly influence hypertrophy outcomes
Sex differences in injury rates and neuromuscular training	Zemkova, E., Hamar, D	To examine sex differences in neuromuscular performance and injury rates in athletes	Cross-sectional study on male and female athletes, focusing on injury rates and neuromuscular adaptation differences	Male and female athletes for sex-based differences in performance and injury	Sex differences in neuromuscular performance are subtle but significant
Training-induced fatigue and recovery in trained vs. untrained individuals	Raikova, R., et al.	To compare fatigue and recovery profiles in trained and untrained individuals	Experimental study comparing fatigue recovery protocols in elite and recreational athletes	Trained vs. untrained individuals comparing fatigue and recovery profiles	Trained athletes recover faster from resistance training-induced fatigue than untrained
Adaptations in muscle fiber type following resistance training	Verbrugge, S.A., et al.	To explore muscle fiber type shifts with long-term resistance training in athletes	Cross-sectional study comparing fiber-type distributions between elite and recreational athletes	Athletes experiencing muscle fiber type shifts due to resistance training	Resistance training induces significant shifts in muscle fiber types in athletes
Differences in resistance training performance between novice and elite athletes	Latella, C., et al.	To compare resistance training performance between novice and elite athletes	Experimental design analysing performance metrics between novice and elite athletes	Novice vs. elite athletes comparing resistance training performances	Elite athletes exhibit superior resistance training performance compared to novices
Resistance training effects on maximal strength and muscle hypertrophy	Lacio, M., et al.	To investigate resistance training effects on muscle hypertrophy and strength in different athlete groups	Systematic review on muscle strength and hypertrophy across various resistance training regimens in trained vs. untrained athletes	Athletes undergoing resistance training for hypertrophy and strength	Resistance training significantly enhances both muscle hypertrophy and strength in athletes

This transparent approach strengthens the credibility of the review’s findings and enables a more nuanced critique of the literature, particularly regarding training specificity, adaptation variability, and the translational gap between empirical research and applied practice in sports settings.

## 2 Neuromuscular adaptations and their significance

Neuromuscular adaptations refer to the complex interplay of muscular and neural changes that enhance force production and athletic performance (Bandy et al., 1990). From a muscular perspective, resistance training induces hypertrophy—an increase in muscle fiber size—and alters muscle architecture, including changes in fiber pennation angle and fascicle length (Harris et al., 2023). These structural adaptations contribute to greater maximal force and power output (Jenkins et al., 2017). However, strength gains are not solely muscular; neural adaptations, such as improved motor unit recruitment and firing rates, also play a critical role in increasing voluntary force production (Bernárdez-Vázquez et al., 2022; Hughes et al., 2018). Importantly, these adaptations have functional relevance—enhancing strength, power, and fatigue resistance, which are key to improving athletic performance and reducing injury risk (Bernárdez-Vázquez et al., 2022). Despite this understanding, the relative contribution of muscular versus neural factors remains contested, especially when comparing elite and recreational athletes, highlighting the need for more targeted investigation.

**FIGURE 1:**
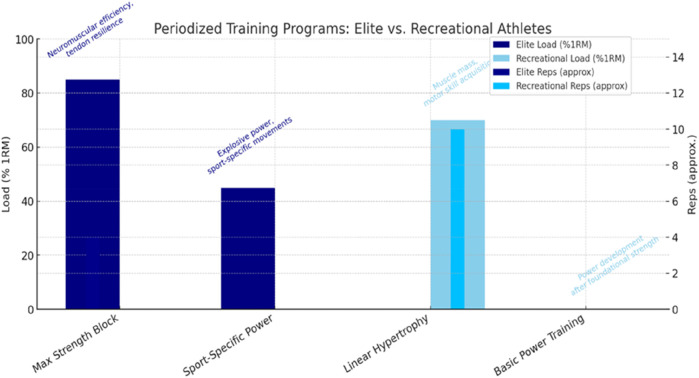
Periodized training programs for elite vs. recreational athletes. Divergent periodization models for elite and recreational athletes. Elite programs emphasize multi-model periodization (strength → power → peaking), while recreational programs focus on linear progression (hypertrophy → strength).

Early-phase strength gains in novice athletes are primarily driven by neural adaptations, as the nervous system becomes more efficient at recruiting motor units and coordinating muscle activation before significant hypertrophy occurs (Bernárdez-Vázquez et al., 2022; Walker et al., 2016). Over time, as training progresses, muscular adaptations—such as hypertrophy and changes in muscle architecture—become the dominant contributors to strength improvements (Faigenbaum and Myer, 2010). For elite athletes, however, neuromuscular adaptations approach their physiological ceiling, requiring novel or more intense stimuli to elicit further gains (May et al., 2022). In contrast, untrained or recreational athletes possess a larger adaptive reserve, allowing them to respond rapidly to even basic training protocols due to their low initial training status (Mangine et al., 2015). This highlights a critical implication that a uniform training approach across different athlete populations is fundamentally flawed (see Figure 1). Elite athletes require highly individualized, progressive overload strategies to bypass adaptive plateaus, whereas novices benefit from foundational strength training focused on neural learning and motor pattern efficiency (Chernozub et al., 2024; Latella et al., 2019). Furthermore, chronic neural adaptations—central to sustained strength gains in trained athletes—eventually plateau, necessitating strategic variation and periodization to sustain progress (Santos et al., 2023). Thus, understanding the distinct neuromuscular trajectories of elite and recreational athletes is essential for designing effective, evidence-based resistance training programs that maximize performance outcomes while minimizing injury risk.

Table 1 presents a synthesis of key studies exploring neuromuscular adaptations to resistance training in elite and recreational athletes. The studies vary in design, participant characteristics, and methodological approaches, examining factors such as muscular recovery, neurophysiological adaptations over time, and the effects of different training modalities (e.g., power training, sprinting) and environmental conditions (e.g., altitude). A consistent trend emerges: elite athletes exhibit slower and smaller neuromuscular gains due to their proximity to physiological ceilings, necessitating more complex and targeted training strategies to stimulate further adaptations. In contrast, recreational athletes demonstrate more rapid and pronounced improvements, attributable to their lower initial fitness levels and greater adaptive capacity. The evidence underscores that optimal training loads and recovery protocols must be tailored to the athlete’s training status, as the same stimulus elicits distinct neuromuscular responses in elite versus recreational populations. This distinction reinforces the need for individualized periodization models to maximize adaptation efficiency and athletic performance while mitigating fatigue and injury risk.

## 3 Resistance training methodologies in elite vs. recreational athletes

Recent research has explored diverse resistance training methodologies to examine neuromuscular adaptations in elite and recreational athletes. Key training variables—load, volume, frequency, exercise selection, rest intervals, and contraction type—are critical for optimizing adaptations (Lorenz et al., 2013; Lacio et al., 2021; Makaruk et al., 2024). Load magnitude significantly impacts outcomes: heavy loads (≥80% 1 RM) maximize strength, while moderate loads (70%–85% 1 RM) are more effective for hypertrophy (Lorenz et al., 2013; Alix-Fages et al., 2022). A recent review confirmed that moderate-to-high loads produce greater strength gains than light loads in both trained and untrained young men (Alix-Fages et al., 2022). However, when training to muscle failure, hypertrophy can occur across a broad load range (30%–90% 1 RM), challenging the traditional load-intensity paradigm (Lorenz et al., 2013). Recreational athletes respond rapidly due to greater adaptive reserves, while elite athletes require higher load specificity and periodization to drive further gains. These findings highlight the need for tailored training protocols, as the same stimulus produces different neuromuscular responses based on training status and adaptation potential (see Table 2).

**TABLE 2:** Training Methodologies for Elite vs. Recreational Athletes.

Training methodology	Elite athletes	Recreational athletes
Program Type	Periodized programs, sport-specific, multi-modal training	Basic resistance training with progressive overload
Training Volume	Higher weekly training volume (e.g., 620 min/week)	Lower weekly training volume (e.g., 155 min/week)
Focus	Strength, power, explosive movements, sport-specific	Hypertrophy, general strength, endurance
Rest Intervals	Shorter rest periods for power and explosive training	Longer rest periods, moderate intensity
Progression	Advanced periodization, frequent variation of exercises	Linear progression, simple overload strategies

### 3.1 Methodologies in elite vs. recreational groups

Studies highlight distinct differences in resistance training methodologies between recreational and elite athletes. Recreational trainees typically follow linear programs with basic lifts, moderate loads, and progressive overload—effective for rapid early gains due to low training baselines (Lopez et al., 2020). Elite athletes, however, engage in periodized programs integrated into sport-specific training, often incorporating high-load, explosive, or specialized exercises (e.g., plyometrics, Olympic lifts) to drive further adaptations (Lorenz and Morrison, 2015; Phillips, 2016). Power training (low-load, high-velocity) combined with heavy strength training enhances force production and rate of force development—critical for elite performance. A meta-analysis by Behm et al. (2017) confirmed that untrained youth achieve larger training effects than trained individuals, reinforcing that novices benefit from simpler programs, while elite athletes require varied and intense stimuli for continued progress (Faigenbaum and Myer, 2010; Guo and Qiao, 2023). This distinction highlights the need for tailored training approaches—progressive overload for novices versus periodized, multimodal strategies for elite athletes—to optimize performance and prevent stagnation.

### 3.2 Common themes

Training methodologies in resistance training literature consistently reflect the principles of specificity and individualization, yet key differences emerge between elite and recreational athletes. Recreational athletes in experimental settings typically follow standardized routines (e.g., 3 days/week, 8–12 reps) to facilitate compliance and controlled outcome measurement—an approach that ensures methodological consistency but may limit ecological validity (Granacher et al., 2018). In contrast, elite athletes’ training often involves higher weekly volume and sport-specific lifts, reflecting the greater complexity of elite development programs (Granacher et al., 2018). A study reported that young elite sports school students averaged 620 min/week of training versus 155 min/week among non-athletic peers, with no adverse effects on growth or academics (Guo and Qiao, 2023; Thiele et al., 2020). However, this raises questions about long-term load tolerance and the potential for overtraining, particularly as elite training often exceeds recommended youth guidelines (Granacher and Borde, 2017). These distinctions underscore that while core training principles apply universally, differences in training dose (volume, intensity) and type (strength versus power) between elite and recreational athletes fundamentally shape neuromuscular adaptations. Greater attention to these contextual factors is crucial for interpreting adaptation outcomes and designing targeted interventions.

## 4 Muscle hypertrophy and architectural adaptations

### 4.1 The magnitude of hypertrophy

A consistent pattern in the literature (See Table 3) indicates that recreational individuals exhibit greater relative hypertrophy in response to resistance training compared to trained athletes (Jenkins et al., 2017; Currier et al., 2023; Kessinger et al., 2020). Novices, starting from a lower baseline, respond robustly to even moderate stimuli, showing significant increases in muscle fiber size and cross-sectional area (Kessinger et al., 2020; Baker et al., 2013). In contrast, trained athletes experience smaller hypertrophic responses, requiring greater stimulus intensity and volume to elicit meaningful gains—a reflection of the principle of diminishing returns (Guo and Qiao, 2023; Reggiani and Schiaffino, 2020; Townsend, 2022). A meta-analysis noted that strength and power gains were significantly larger in untrained youth than in trained adolescents, underscoring greater adaptive capacity in novices (Guo and Qiao, 2023). Interestingly, studies show that untrained individuals can hypertrophy with minimal volume (e.g., a single set), whereas trained lifters require higher volumes for continued muscle growth (Reggiani and Schiaffino, 2020; Townsend, 2022). This highlights the critical need for progressive overload and training variation, especially in elite athletes, where adaptation thresholds are higher and additional muscle growth becomes increasingly difficult to achieve.

**TABLE 3:** Neuromuscular Adaptation in Elite vs. Recreational Athletes.

Neuromuscular factor	Elite athletes	Recreational athletes
Muscle Hypertrophy	Smaller relative gains, requires higher volume/intensity	Larger relative gains in early training phases
Neural Drive	Higher maximal voluntary activation, refined coordination	Rapid early improvements, but lower ceiling
Motor Unit Recruitment	More efficient activation, lower antagonist co-contraction	More inefficient recruitment, higher co-contraction
Muscle Fiber Shifts	More Type II fibres retained, possibly hyperplasia effects	Type IIx - IIa transition more pronounced
Fatigue & Recovery	Faster neuromuscular recovery, accustomed to high loads	Slower recovery, more fatigue from lower loads

### 4.2 Differences in muscle architecture

Long-term resistance training induces not only muscle hypertrophy but also architectural modifications, enhancing force transmission and functional output (Schoenfeld et al., 2017; Braz et al., 2022; Fukutani and Kurihara, 2015; Guex et al., 2016). Increased fascicle length and pennation angle are common adaptations, with power-trained athletes often displaying longer fascicles suited for high-speed contraction (Martin-Rodriguez et al., 2024; Cooper et al., 2021). Notably, elite strength-trained individuals exhibit distinct muscle architecture compared to untrained peers (Davis et al., 2020). A recent biopsy study reported that trained adults possess both larger and more numerous muscle fibers in the biceps than untrained controls, suggesting hyperplasia or fiber retention with training (Zaras et al., 2022). This finding challenges the long-standing notion that hypertrophy alone drives muscle enlargement, highlighting a potential role for fiber recruitment and preservation (Maeo et al., 2024; Maughan et al., 1984). Moreover, trained muscles exhibit increased myofibrillar density and tighter filament packing, indicative of ultrastructural remodelling in response to chronic high-load training (Zaras et al., 2022). These architectural refinements suggest that elite athletes’ muscles are not only larger but also structurally optimized for greater force production—an adaptation that may confer a competitive edge.

### 4.3 Resistance training modalities differentially influence muscle architecture

Resistance training differentially alters muscle architecture depending on the type and velocity of contraction. Heavy-load strength training (e.g., ≥80% 1RM, slow velocity) typically increases pennation angle by around 15%–20%, enhancing force capacity through a larger physiological cross-sectional area (Fukutani and Kurihara, 2015). However, fascicle length remains relatively unchanged, as hypertrophy occurs primarily via radial growth (Braz et al., 2022).

Conversely, power-oriented training (e.g., ballistic or plyometric protocols with moderate loads and high velocity) promotes fascicle elongation around 8%–12%, which supports faster contraction speeds and greater movement efficiency (Guex et al., 2016). In such cases, increases in pennation angle are modest 5%–10% (Plotkin et al., 2021).

These adaptations are context-specific whereby elite athletes show discipline-specific remodelling, for example, longer fascicles in sprinters, greater pennation in powerlifters, while recreational trainees typically exhibit generalized changes (Zaras et al., 2022). Practically, aligning training modalities with desired architectural outcomes is critical for performance optimization, particularly in high-performance settings where specificity can influence adaptation ceilings.

### 4.4 Plateaus and potential limits

Hypertrophy in elite athletes often reaches a plateau without novel stimuli, highlighting the importance of periodization strategies alternating hypertrophy-focused (high-volume) and strength/power phases to sustain progress (Lopez et al., 2020; Antonio and Gonyea, 1993). Genetic factors, such as muscle fiber count and satellite cell responsiveness, may partly explain interindividual differences in hypertrophic potential and why some athletes exhibit superior muscle development (Gelman et al., 2022; Verbrugge et al., 2018). This underscores the need for personalized training protocols tailored to genetic predispositions and training history. While recreational athletes typically experience rapid initial hypertrophy due to low baseline fitness, elite athletes encounter diminishing returns, requiring complex and varied stimuli to achieve further gains (Reggiani and Schiaffino, 2020). Importantly, longer-term studies are needed to clarify the ceiling of muscle adaptation in elite populations and whether targeted interventions can extend this limit. Moreover, the distinct architectural features observed in elite muscles reflect specialized adaptations to chronic training stress—an outcome that may be as much a product of genetic endowment as it is of training specificity.

## 5 Neural adaptations: motor unit recruitment and neural drive

### 5.1 Early-phase neural gain

Neural adaptations are a defining feature of early strength gains, especially in recreational trainees. In the initial weeks of resistance training, untrained individuals enhance motor unit recruitment and firing efficiency, as reflected by increased electromyographic (EMG) activity during maximal efforts and improved agonist-antagonist coordination—changes that occur before measurable hypertrophy (Bazgir et al., 2017; Grgic and Schoenfeld, 2018). Classic studies have long established that novices can improve strength through enhanced motor unit activation and synchronization, even without muscle size increases (Häkkinen et al., 1998) (Raikova et al., 2021). Recent findings reinforce this, with a 2023 systematic review confirming that trained individuals exhibit higher maximal voluntary activation compared to novices, reflecting superior neural efficiency (Santos et al., 2023). This underscores the role of repeated neural training in refining motor unit recruitment patterns, which partly explains why elite athletes achieve greater contractile force efficiency. However, the extent to which neural adaptations plateau in elite populations remains underexplored, raising the question of whether targeted neural training could further enhance performance in already highly trained athletes.

### 5.2 Motor unit recruitment and firing rate

Highly trained athletes exhibit refined motor unit recruitment patterns, contributing to superior neuromuscular efficiency. A systematic review confirmed that long-term trained individuals generate submaximal force with lower muscle activity than untrained individuals, suggesting that trained muscles require fewer motor units or lower firing rates to produce the same absolute force (Grgic and Schoenfeld, 2018; Häkkinen et al., 1998). This efficiency enables greater endurance and reduced fatigue at submaximal loads (Hou et al., 2021). Furthermore, trained athletes demonstrate reduced antagonist co-contraction during movements, enhancing net force production (Hou et al., 2021; Zemková and Hamar, 2018). For instance, in a trained weightlifter performing an overhead press, triceps (agonist) activation is high while biceps (antagonist) activation remains minimal, allowing more efficient force transmission (Neto et al., 2019; Farias et al., 2017). In contrast, untrained individuals often co-contract antagonists unintentionally, reducing efficiency and increasing energy expenditure (Zemková and Kováčiková, 2023; Enoka and Duchateau, 2008). This highlights a key neuromuscular adaptation from training—improved motor coordination and inhibition of antagonistic activity—which distinguishes trained from novice athletes (see Table 4). However, whether these adaptations plateau with prolonged training remains an open question, warranting further exploration.

**TABLE 4:** Key differences in neural adaptation between elite and recreational athletes.

Neural adaptation	Elite athletes	Recreational athletes
Maximal Voluntary Activation	Higher activation capacity, refined control	Lower activation capacity, requiring more effort for maximal force
Motor Unit Recruitment	More efficient, using fewer motor units for high force	Less efficient, needing more motor units for same output
Antagonist Co-contraction	Lower antagonist activation, more efficient movement	Higher antagonist co-contraction, less efficient movements
Neural Efficiency	Greater neural efficiency for submaximal effort	Lower neural efficiency, requiring higher effort per rep
Coordination	Fine-tuned coordination across muscle groups	Less coordinated movement patterns during complex lifts

### 5.3 Intermuscular coordination

Intermuscular coordination, the synchronized activation of multiple muscle groups during complex movements, is a critical yet underexplored aspect of neuromuscular adaptation. Skilled athletes exhibit superior intermuscular coordination, allowing for more efficient and powerful execution of complex lifts (Akbar et al., 2022; Wang et al., 2022; Wang et al., 2024). Research highlights that long-term training enhances this coordination, but the underlying mechanisms remain poorly defined (Santos et al., 2023). For example, a novice learning to squat typically displays poor motor sequencing, with quadriceps, glutes, hamstrings, and trunk muscles firing sub optimally (Iacoangeli, 2022). In contrast, an elite powerlifter demonstrates refined muscle activation patterns, optimizing force transmission and minimizing energy waste—an outcome of years of neural refinement (Sale, 1988; Iacoangeli, 2022; Gabriel et al., 2006). This implies that intermuscular coordination is a neural adaptation specific to practiced movements, reinforcing the importance of sport-specific training. Nevertheless, the extent to which coordination improvements plateau in elite athletes remains unclear, highlighting the need for more targeted research on neural plasticity and intermuscular synergies at advanced training levels.

### 5.4 Neural adaptation plateaus

Similar to muscular gains, neural adaptations plateau over time, with the ceiling often reached earlier in trained athletes (Hughes et al., 2018; Gelman et al., 2022; Balshaw et al., 2016). Once near-maximal motor unit recruitment is consistently achieved, further strength improvements depend more on muscle hypertrophy or advanced neural strategies (e.g., increased firing rates, reduced antagonist co-activation) (Gabriel et al., 2006). Innovative training methods (e.g., explosive or inhibitory control drills) have been proposed to stimulate further neural gains once recruitment efficiency is maximized, but evidence supporting their long-term efficacy remains limited (Santos et al., 2023; Gabriel et al., 2006). Methodological challenges also complicate research in this area. Surface EMG studies comparing elite and novice individuals are prone to signal amplitude bias due to differences in muscle size, which complicates straightforward interpretation of neural efficiency (Škarabot et al., 2021b). This highlights a critical limitation in current methodologies and underscores the need for more refined approaches to isolate genuine neural changes. Ultimately, while neural drive and coordination distinguish elite performers, optimizing these adaptations requires increasingly sophisticated and individualized training strategies at advanced levels.

### 5.5 Rate of force development (RFD) adaptations

The rate of force development (RFD) as the speed at which force is produced is a key determinant of explosive athletic performance. Training status and modality elicit phase-specific RFD adaptations that are functionally relevant across sport contexts.

Early-phase RFD (0–100 ms) primarily reflects neural drive and is significantly enhanced by power-focused modalities such as plyometrics and Olympic lifts. These interventions improve motor unit recruitment thresholds and firing frequency, particularly in high-threshold motor units (Aagaard et al., 2002). Elite athletes typically display 20%–30% higher early-phase RFD than recreational counterparts, attributed to more efficient spinal reflex pathways and Ia-afferent feedback (Maffiuletti et al., 2016).

Late-phase RFD (100–200 ms) is more influenced by morphological adaptations, including increased tendon stiffness and contractile protein efficiency, typically resulting from high-load strength training (Balshaw et al., 2016). Recreational trainees often exhibit greater relative improvements in late-phase RFD (+40–60%), reflecting their lower neuromuscular baseline (Jenkins et al., 2017). Notably, elite athletes often prioritize early-phase RFD for task-specific explosiveness, for example, sprint starts, whereas novices benefit from foundational late-phase improvements (Walker et al., 2016). These distinctions underscore the importance of tailoring resistance training strategies to optimize neural adaptation trajectories based on the athlete’s performance level and sport-specific demands.

## 6 Muscle fiber type transitions and genetic factors

### 6.1 Fiber type composition

Human skeletal muscle fibers range from slow-twitch (Type I) to fast-twitch (Type II), with Type IIa fibers being fast and moderately fatigue-resistant, while Type IIx fibers are faster but highly fatigable (Plotkin et al., 2021; Talbot and Maves, 2016). Training status significantly influences this distribution (Hall et al., 2021). Untrained young individuals typically present a balanced Type I and II fiber mix with a minor IIx fraction (Murach et al., 1985). Resistance training consistently shifts the profile from IIx toward IIa, enhancing fatigue resistance while preserving high-force output (Plotkin et al., 2021). Notably, this transition occurs early in training; several studies report a rapid decline in IIx proportion as fibers convert to IIa under repeated activation (Trappe et al., 2006). Sprint or power training tends to reinforce this IIa dominance at the expense of IIx (Plotkin et al., 2021). However, the near-universal decline in IIx with training (regardless of strength or endurance focus) suggests that muscle plasticity leans toward improved oxidative efficiency over maximal speed, which may reflect an evolutionary trade-off rather than a purely performance-driven adaptation.

**FIGURE 2:**
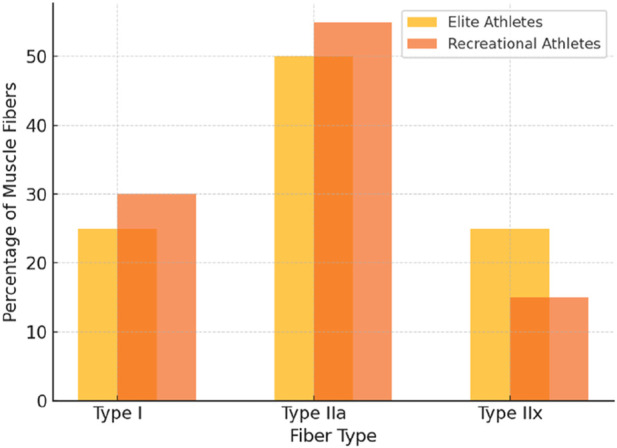
Muscle fiber type distribution (Elite vs. Recreational athletes).

Long-term trained athletes sometimes display fiber-type distributions that diverge from short-term patterns (see Figure 2). Cross-sectional studies have reported that some elite power athletes retain or even increase Type IIx fibers, contrary to the typical IIx-to-IIa shift observed in early training (Plotkin et al., 2021). Notably, an analysis of competitive bodybuilders revealed a higher proportion of Type IIx fibers (∼15%) compared to untrained controls (∼5%), despite similar IIa fiber percentages (∼45%) and reduced Type I content (D'Antona et al., 2006). This finding challenges the widely accepted view that IIx fibers inevitably transition to IIa with resistance training. It raises the possibility that intense, high-load training (combined with genetic predisposition) might enable the preservation or re-expression of IIx fibers over time. Similarly, an elite sprinter was documented with an unusually high IIx content (24%), suggesting that individuals genetically endowed with explosive muscle fibers may resist the IIx-to-IIa shift under specialized training (Plotkin et al., 2021; Trappe et al., 1985). However, the extent to which training versus genetic factors drive this divergence remains unclear and warrants further investigation (Flück et al., 2019; Simoneau and Bouchard, 1995).

### 6.2 Role of genetics

Genetic factors exert a substantial influence on neuromuscular adaptation. Fiber-type distribution is partly hereditary, i.e., individuals with a higher proportion of Type II fibers often excel in power sports, while those with more Type I fibers tend to perform better in endurance events (Tesch and Karlsson, 1985; Serrano et al., 2019). Although training can modify fiber characteristics to some extent (phenotypic plasticity), genetic limits are evident. Some disparities between elite and recreational athletes may reflect pre-existing differences rather than training-induced changes, raising the issue of selection bias whereby those who naturally respond well to training are more likely to reach elite status. Furthermore, genetic factors such as hormonal profiles, tendon insertions, and neural efficiency can influence training responsiveness. Research into gene variants (e.g., ACTN3, ACE) has sought to explain individual differences in strength and power adaptations, but the effect sizes are generally small and inconsistent (Pickering and Kiely, 2017; Gineviciene et al., 2016). This suggests that while genetic predisposition shapes the adaptive ceiling, training determines how close one gets to that limit. For instance, a fast-twitch-dominant individual will likely never convert to a predominantly slow-twitch profile, regardless of training, and *vice versa* for slow-twitch-dominant individuals.

From a practical standpoint, recreational athletes typically show a predictable shift from Type IIx to IIa fibers with training, enhancing fatigue resistance while preserving force capacity. Elite athletes, in contrast, exhibit fiber-type distributions shaped by both specialized training and genetic inheritance. This underscores the importance of individualized training approaches. In this light, power-oriented athletes may require higher-intensity, shorter-duration work, whereas endurance-focused athletes might benefit from more oxidative-based training. Future exploration into genetic testing and fiber typing could refine these strategies, but current evidence suggests that genetic predisposition remains a limiting factor in athletic specialization and adaptation (Plotkin et al., 2021).

## 7 Fatigue, recovery, and injury risk differences

### 7.1 Fatigue and recovery in men vs. women

Sex is a significant factor in neuromuscular performance and recovery, but the underlying mechanisms and practical implications remain complex. On average, men demonstrate greater absolute strength and power than women, primarily due to larger muscle mass and a greater cross-sectional area of Type II fibers (Santos Junior et al., 2024; Hunter, 2016; Miller et al., 1993; Nuzzo, 2023). Conversely, women often exhibit greater fatigue resistance during submaximal isometric tasks, which is attributed to smaller muscle fiber size (reducing blood flow occlusion) and a higher reliance on oxidative metabolism (Santos Junior et al., 2024). However, the evidence regarding sex differences in post-exercise recovery is inconsistent.

A recent narrative review by Nuzzo (2023) concluded that no significant sex differences exist in strength loss and muscle soreness following muscle-damaging exercise, suggesting that men and women of similar training status recover comparably from intense resistance training (Nuzzo, 2023). This challenges earlier assumptions that women consistently recover faster. However, conflicting evidence exists. In this regard, a study on trained young adults reported that women experienced a more prolonged drop in performance (e.g., countermovement jump height and concentric strength) 24–72 h post heavy squat workout compared to men (Davies et al., 2018). Specifically, 24 h post-exercise, women’s jump height declined by 20% versus 10% in men, a fact highlighting a notable disparity in explosive recovery (Davies et al., 2018). This raises the possibility that sex differences in recovery may depend on the type of performance metric assessed (e.g., maximal force vs. explosive power) and the specific exercise protocol used.

Practically, this variability suggests that recovery strategies should be individualized rather than generalized by sex. The greater fatigue resistance observed in women during submaximal tasks may not translate into faster recovery from high-intensity, explosive efforts as a nuance often overlooked in training programs. Additionally, elite athletes, regardless of sex, are more likely to experience greater muscle damage due to higher training loads, reinforcing the need for tailored recovery protocols. Future research should clarify whether sex-based differences in recovery reflect intrinsic physiological differences or are influenced by training background and hormonal profiles. Until then, coaches and practitioners should monitor individual recovery patterns rather than apply blanket assumptions based on sex.

### 7.2 Central vs. peripheral fatigue

Resistance training elicits both central fatigue (reduced neural drive from the central nervous system) and peripheral fatigue (impairments within the muscle itself), with responses differing markedly by training status. To clarify, elite athletes typically exhibit attenuated peripheral fatigue due to superior mitochondrial density, capillarization, and oxidative enzyme activity, which enhance local muscle endurance and accelerate recovery (Enoka and Duchateau, 2008). Their training also upregulates muscle repair pathways, enabling better tolerance to high-volume loads (Bazgir et al., 2017). Central fatigue in elite populations is minimized through enhanced motor unit synchronization, corticospinal excitability, and neural efficiency, contributing to sustained performance under repeated exertion (Raikova et al., 2021).

Conversely, recreational athletes experience more pronounced peripheral fatigue including higher CK levels, delayed-onset muscle soreness due to lower baseline conditioning and slower metabolic adaptation (Halson, 2014). Early-phase training often induces central fatigue, driven by suboptimal motor unit recruitment and elevated perceived exertion (Škarabot et al., 2021b). Over time, resistance training improves both fatigue resistance and recovery kinetics, but elite athletes consistently demonstrate 30%–50% faster recovery post-exercise, facilitating greater training frequency and intensity (Andersson et al., 2008). These distinctions underscore the need for training programs to tailor fatigue management strategies to athlete experience level, optimizing adaptation while minimizing injury risk.

### 7.3 Training-induced fatigue in trained vs. untrained

**FIGURE 3:**
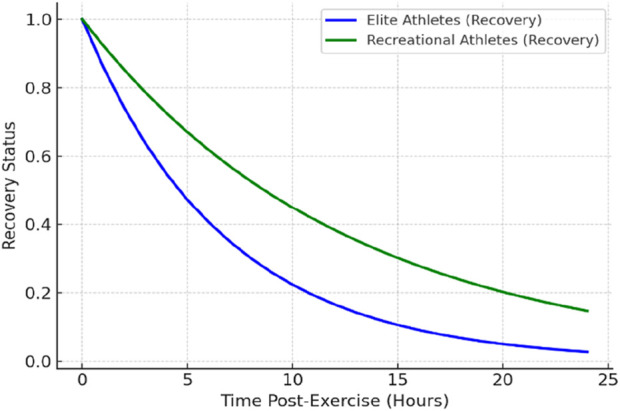
Fatigue and Recovery Trends in Elite vs. Recreational Athletes.

Training status significantly influences fatigue and recovery dynamics (see Figure 3), with marked differences observed between untrained individuals and elite athletes. Untrained individuals often experience pronounced delayed onset muscle soreness (DOMS) and substantial reductions in force output following novel exercise (an effect known as the “first bout” phenomenon) (Halson, 2014). However, they typically exhibit the repeated bout effect, wherein subsequent exposures to the same exercise induce progressively less muscle damage due to rapid neuromuscular adaptations (McHugh, 2003). This early-stage adaptation necessitates longer recovery periods initially, but within weeks, novice trainees display significantly improved recovery efficiency (Nosaka and Aoki, 2011).

In contrast, elite athletes, having undergone prolonged training exposure, demonstrate a blunted muscle damage response to familiar exercises. Their neuromuscular systems are highly conditioned to recover efficiently from standard training stimuli. Nonetheless, given their higher absolute training loads, elite athletes may still face recovery challenges and thus implement advanced recovery strategies (e.g., precise nutrition, optimized sleep, and active recovery techniques) to maintain performance (Halson, 2013).

Another key distinction lies in intra-session neuromuscular fatigue resistance. Trained individuals can sustain a higher training volume at near-maximal intensities before experiencing significant fatigue. This is largely attributed to greater neuromuscular efficiency that allows them to generate force with reduced motor unit recruitment relative to their maximum (Akbar et al., 2022; Lyons et al., 2006; Garrett et al., 2023). Consequently, trained athletes can tolerate higher training frequencies (e.g., 4–6 sessions per week), whereas untrained individuals may require longer recovery periods to prevent excessive neuromuscular strain.

While these distinctions are well-documented, further research is needed to determine whether elite athletes’ superior recovery is solely a result of training adaptations or if inherent physiological factors such as genetic predisposition, hormonal milieu contribute to their resilience. Additionally, most studies focus on short-term recovery markers like soreness, force output, whereas long-term fatigue accumulation (over weeks or months) remains underexplored. Future research should examine whether highly trained individuals experience subtle chronic fatigue despite their apparent rapid recovery in acute phases.

### 7.4 Injury risk considerations

Injury risk in resistance training is generally low when appropriate technique, progression, and recovery strategies are implemented. However, distinct risk profiles exist across training populations. Novice trainees are more susceptible to acute injuries including muscle strains and joint sprains due to improper form, excessive load progression, or inadequate adaptation time (Faigenbaum and Myer, 2010). Overuse injuries can also emerge when beginners adopt high-frequency training before their musculoskeletal system has sufficiently adapted. These risks highlight the need for structured progression models, yet many beginners (particularly in self-guided training) lack the necessary supervision to prevent such issues.

Elite athletes, while technically proficient, encounter cumulative stress injuries due to the extreme loads and high training volumes they endure. Chronic tendinopathies, particularly in weightlifters and power athletes, are well-documented consequences of prolonged exposure to high-intensity training (Faigenbaum and Myer, 2010; Mersmann et al., 2017). In adolescents, a developmental imbalance between muscle and tendon strength has been proposed as a risk factor for injury. Notably, Mersmann et al. (Mersmann et al., 2017) reported that young volleyball athletes demonstrated disproportionate quadriceps strength relative to patellar tendon stiffness, predisposing them to tendon-related pathologies (Granacher et al., 2018; Mersmann et al., 2017). This finding raises concerns about whether current strength training paradigms adequately address tendon adaptation, particularly in youth programs. Strategies such as isometric holds and slow eccentrics have been recommended to enhance tendon robustness, yet their integration into conventional training remains inconsistent. Future research should explore optimal programming for tendon conditioning in young athletes to mitigate long-term injury risks.

Sex-based differences in injury risk further complicate resistance training safety. While men sustain higher absolute strength-training injuries (potentially due to heavier loads and risk-taking behaviors) women face increased susceptibility to specific joint injuries, particularly ACL tears (Nuzzo, 2023). These disparities are often attributed to anatomical and hormonal factors, yet a growing body of evidence supports neuromuscular training interventions (e.g., plyometrics, proprioceptive drills, and strength training) as effective injury-prevention strategies. A meta-analysis demonstrated that structured neuromuscular programs could reduce lower extremity injury risk by approximately 42%, particularly when performed 2–3 times per week for >30 min per session over several months (Granacher et al., 2018; Steib et al., 2017). Despite this compelling evidence, the adoption of such protocols remains inconsistent across sports organizations and youth development programs.

As implications for training and policy, the intersection of injury risk, training adaptation, and recovery management underscores the necessity for population-specific strength training guidelines. While elite athletes demonstrate superior neuromuscular efficiency and recover more effectively from submaximal work, their high-intensity workloads demand meticulous recovery strategies to prevent overuse syndromes (Andersson et al., 2008). Correspondingly, while men and women exhibit more similarities than differences in overall training adaptations, subtle distinctions in fatigue resistance, power recovery, and injury susceptibility necessitate nuanced programming (Lewis et al., 1986).

Ultimately, evidence-based resistance training frameworks that emphasizes progressive overload, movement quality, supervised technique development, and recovery protocols are essential to balancing performance enhancement with injury risk mitigation. The widespread integration of injury prevention strategies into youth and elite training systems remains a critical yet underemphasized area, warranting further policy-driven implementation in sports organizations, schools, and professional training environments.

## 8 Methodological strengths and weaknesses in the literature

Research on neuromuscular adaptations in elite versus recreational athletes faces key methodological challenges, particularly in study design and participant selection. Cross-sectional studies reveal end-state differences in muscle fiber composition and neural activation between trained and untrained groups (Maeo et al., 2024; MacDougall et al., 1984; Sale et al., 1987). However, they cannot establish causation or the timeline of adaptations, and are prone to self-selection bias whereby elite athletes may possess favorable genetic traits or early training advantages that the untrained group lacks, meaning that observed differences may precede training rather than result from it. Longitudinal studies better capture cause-effect relationships but are typically short-term (6–12 weeks), limiting insights into long-term adaptations that extend beyond the scope of typical study durations. Few studies track athletes over years due to feasibility issues, leaving a gap in understanding elite-level adaptations. Additionally, most studies are conducted in controlled settings, limiting ecological validity. Future research should adopt multi-year designs and mixed-method approaches to capture the complexity of elite neuromuscular adaptations.

### 8.1 Training interventions and controls

Designing effective training protocols for longitudinal studies presents significant challenges due to the variability in exercise selection, intensity, and volume across studies, complicating direct comparisons. Additionally, authentic randomization in elite athletes remains rare, as assigning them to suboptimal training is impractical and ethically questionable. Consequently, most intervention studies focus on recreational or untrained subjects, skewing insights toward novice adaptation rather than elite-level responses. For example, a systematic review on training load reported that only 19% of participants were classified as trained (Lacio et al., 2021), highlighting a critical gap in evidence for trained lifters.

Blinding is also impossible in exercise studies since participants are aware of their training protocols and often the study hypothesis, introducing potential placebo and motivation biases. This raises concerns about the internal validity of many findings. Furthermore, reliance on small sample sizes and expert opinion in elite athlete research weakens the generalizability of conclusions. To address these limitations, future research should prioritize standardized protocols and explore innovative designs such as crossover or mixed-method approaches to generate more reliable and transferable insights for advanced athletes.

### 8.2 Measurement techniques

Assessing neuromuscular adaptations involves diverse techniques, each with specific limitations. Imaging methods like MRI and ultrasound provide valuable insights into muscle size, but EMG measurements can be confounded by muscle impedance and hypertrophy, requiring careful normalization. A recent study cautioned against attributing EMG changes purely to neural adaptations due to the confounding effect of muscle growth (Škarabot et al., 2021b). Muscle biopsy analysis also presents sampling limitations, as it reflects only a small portion of the muscle, which may not accurately represent whole-muscle characteristics although emerging imaging methods aim to address this (Maeo et al., 2024). Strength testing is similarly influenced by test familiarity; elite athletes may perform better due to greater experience with specific movement patterns. While familiarization sessions for untrained participants aim to reduce this bias, they are not always standardized across studies, limiting comparability.

Sample characteristics present additional inconsistencies. Definitions of “elite” vary, with some studies requiring national or professional competition status, while others consider greater than 1 year of resistance training sufficient. This inconsistency complicates direct comparisons. Furthermore, research remains male-dominated; a review noted that female athletes are often underrepresented in strength training studies (Suchomel et al., 2016), and even when included, small sample sizes weaken statistical power for sex-specific insights. Age definitions are similarly inconsistent whereby “young” can range from adolescence to mid-30 s, despite clear developmental differences in neuromuscular adaptation. Greater consistency in defining training status, sex, and age across studies is essential for improving the reliability and applicability of findings.

### 8.3 Quality and bias

The methodological quality of research on neuromuscular training adaptations is generally moderate to high, but notable limitations persist. For instance, a systematic review on training load reported that while most included studies were of moderate-to-high quality, only 2 out of 23 scored above 6 on a quality scale (Lacio et al., 2021). This highlights a recurring issue with study rigor. Small sample sizes (<15 per group) remain common due to the logistical demands of training studies, particularly in elite settings. The absence of control groups and inconsistent reporting of randomization and assessor blinding further weakens internal validity, increasing the risk of bias and inflating effect sizes.

A strength in recent years is the increased use of meta-analyses, which help identify consistent patterns across studies and reduce reliance on individual study outcomes. For example, meta-analytic evidence has reinforced the conclusion that higher training loads produce greater strength gains (Lacio et al., 2021). But, the generalizability of such conclusions remains limited by the overrepresentation of recreational athletes and young males. The underrepresentation of female athletes and well-trained populations restricts the applicability of findings to broader athletic contexts (Townsend, 2022; Suchomel et al., 2016).

Moreover, differences between elite and recreational athletes are sometimes exaggerated or obscured by study design flaws. Without long-term, well-controlled studies in elite populations, conclusions about training adaptations remain tentative. Addressing these gaps through larger sample sizes, improved randomization, and more diverse participant pools (including female and highly trained athletes) is essential for advancing the field.

## 9 Gaps in the literature and future research directions

Long-term training studies in elite athletes are scarce, limiting understanding of how neuromuscular gains progress and plateau. Future research should focus on multi-year tracking and controlled trials in sub-elite athletes transitioning to elite status. Collaboration with coaches and sports organizations is essential to overcome logistical challenges.

Despite growing interest, female athletes remain underrepresented in neuromuscular adaptation research. Future studies should compare adaptation patterns in men and women across training states to identify potential sex-based differences in hypertrophy, neural changes, and fiber type shifts. Findings could inform sex-specific training guidelines or confirm similar adaptive responses when training is matched.

Limited data exist on how neuromuscular adaptations progress from adolescence to early adulthood. Longitudinal cohort studies tracking youth athletes from puberty to maturity could clarify how training responsiveness evolves with growth and identify critical periods, such as peak height velocity, where targeted training may optimize gains or mitigate injury risk.

The neural mechanisms underlying improved intermuscular coordination and reduced co-contraction remain unclear. Studies using advanced neurophysiological tools like transcranial magnetic stimulation, high-density EMG, could clarify how elite and novice lifters adapt differently. Insights into motor unit recruitment and antagonist suppression may refine strategies to enhance explosive performance.

Evidence suggesting that trained individuals may have more muscle fibers or myofibrils than untrained ones warrant clarification. It remains unclear if this reflects true hyperplasia or fiber retention. Advanced imaging techniques including diffusion-tensor MRI combined with biopsies could confirm whether long-term training increases fiber number or density and under which conditions.

Individual variability in training response remains poorly understood. Research should identify predictors such as genetic markers, fiber type, hormonal, and neural factors to explain why some individuals are high responders while others are not. Exploring tailored interventions like volume adjustments and vascular occlusion to enhance responsiveness, especially in elite athletes, is warranted.

The impact of concurrent training on neuromuscular adaptation in elite athletes remains unclear. Future research should investigate how combining heavy strength training with high aerobic loads influences neural and muscular gains compared to strength training alone, particularly in team sport athletes where concurrent training is common.

Optimal periodization strategies for neuromuscular gains in advanced athletes remain unclear. Research should compare block models, i.e., separate hypertrophy, strength, and power phases with daily undulating models to determine which best enhances neural adaptation. Studies should include both sexes, use standardized protocols, and collaborate with elite sports programs to improve applicability and methodological rigor.

## 10 Practical implications and recommendations for youth athletes’ training

Novice athletes benefit from low to moderate training volumes focused on technique and gradual loading, which can yield substantial early gains while minimizing injury risk. In contrast, elite athletes require individualized, periodized programs with adjusted intensity and volume to sustain progress. Coaches should tailor stimuli to training status, balancing challenge and recovery to optimize adaptation.

Youth athletes should develop a strength base before engaging in high-speed or plyometric training. Strength training has shown large improvements in strength (ES = 1.14), while power training alone yields minimal strength gains. An 8–12-week strength phase can enhance adaptation in novice athletes, while elite youth may benefit from off-season strength blocks to reinforce their foundation.

Injury-prevention exercises should be integral to youth training programs. Neuromuscular training reduces injury risk by 42%, highlighting the value of structured warm-ups focused on balance, landing mechanics, and core strength. Exercises like jump-landings with feedback and single-leg balance drills are particularly beneficial, especially for young female athletes prone to ACL injuries. Sports programs should mandate such protocols.

Balancing high training loads with recovery is crucial for elite youth athletes. Monitoring fatigue through subjective feedback and performance tests can guide adjustments. For example, sustained drops in jump performance post-training may signal inadequate recovery. Implementing deload weeks, promoting sleep and nutrition education, and integrating recovery modalities can optimize adaptations and prevent overtraining.

Men and women show similar relative improvements from resistance training, supporting equal training opportunities. Female athletes should engage in heavy resistance training with proper coaching to build confidence and technique. While women may show slightly smaller gains in explosive power, incorporating high-rate force development exercises or plyometrics can enhance outcomes. Personalizing programs to the individual, rather than generalizing by sex, remains key.

For young elite athletes in growth phases, strengthening tendons alongside muscle development is essential. Isometric holds such as wall sits and slow eccentrics like Nordic curls can enhance tendon stiffness and reduce injury risk. Monitoring tendon pain and adjusting loads around peak height velocity is critical. Encouraging multi-sport participation can also promote balanced development and prevent overuse issues.

Once a strength foundation is established, elite athletes can benefit from advanced techniques to drive further neuromuscular gains. Periodization, complex training, accentuated eccentrics, and blood flow restriction can provide novel stimuli. Coaches should introduce these methods gradually and systematically to assess their effectiveness and avoid overload, ensuring continued adaptation in advanced athletes.

## 11 Policy implementations for youth programs

Organizations should promote supervised resistance training for youth, dispelling outdated myths about stunted growth with evidence showing no adverse effects on development. Schools and sports academies should integrate age-appropriate strength programs led by qualified professionals. Talent development programs might use physiological testing such as jump height and sprint speed to guide training based on individual strengths, ensuring a balanced athletic foundation to prevent burnout or injury. Evidence-based policies that account for training status, biological sex, and developmental stage can help young athletes maximize neuromuscular potential safely and effectively.

## 12 Conclusion

Over the past decade, research has deepened our understanding of how neuromuscular adaptations to resistance training differ between elite and recreational athletes. Novices typically experience rapid hypertrophy and fiber-type shifts, whereas trained individuals face more gradual gains as they approach physiological limits. Neural adaptations which enhanced motor unit recruitment, firing rates, and coordination are key in early training and remain critical for elite performance, though they plateau without advanced stimuli. Young elite athletes often show superior neuromuscular efficiency and muscle morphology but face challenges like training plateaus and muscle-tendon imbalances. Despite progress, gaps remain particularly in understanding sex-specific adaptations and elite cohorts that highlights the need for more inclusive and long-term studies. Training programs should be tailored to an athlete’s experience: foundational programs for novices, periodized and innovative approaches for advanced athletes, and injury prevention strategies for all. Policymakers should promote supervised resistance training in youth, reinforcing its safety and developmental benefits. Neuromuscular adaptations drive strength and power improvements, but training responses are highly individualized, underscoring the importance of evidence-based, tailored programs for maximizing athletic performance.
